# Rechallenge therapy and treatment holiday: different strategies in management of metastatic colorectal cancer

**DOI:** 10.1186/1756-9966-32-92

**Published:** 2013-11-18

**Authors:** Giuseppe Tonini, Marco Imperatori, Bruno Vincenzi, Anna Maria Frezza, Daniele Santini

**Affiliations:** 1 Department of Medical Oncology, University Campus Bio-Medico, Via A Del Portillo 200, 00128, Rome, Italy

**Keywords:** Colorectal cancer, Drug resistance, Rechallenge therapy, Intermittent treatment

## Abstract

Fluoropyrimidines, oxaliplatin, irinotecan and biologic therapies (Bevacizumab, Panitumumab, and Cetuximab) represent the backbone of metastatic colorectal cancer (CRC) treatment. The improvement in survival for mCRC patient led to two main outstanding issues: 1) there is a significant number of patients progressing beyond the third or fourth line of treatment still suitable for further therapy when enrollment into clinical trial is not possible. In this situation, the role of any therapy rechallenge (either chemotherapy alone, chemotherapy and biologic therapy or biologic therapy alone) is still not clear, particularly in patients who had previously responded, and if treatment choice is based on traditional dogma of primary and secondary resistance, rechallenge does not seem to be justified. 2) Prolonged intensive treatment is burdened from the high risk of cumulative toxicity, worsening in quality of life and a not well defined possibility of early acquired resistance.

Different hypothesis could justify the research of different strategy in treatment of mCRC:

1) Epigenetic changes might drive resistance and treatment could induce these changes. Re-expression of silenced tumor suppressive genes might resensitize tumors to therapy. It is therefore possible that a drug holiday (intermittent treatment) could allow reversion to a previous epigenetic profile. Moreover an intermittent treatment could delay acquired resistance. 2) It is plausible that tumor grows as a polyclonal mass. If it responds but then becomes resistant to one or more treatments, retreatment might be successful if changing therapies allows to that clone of cells to re-emerge. On these basis, we focused this review on the actual evidences in management of mCRC patients in terms of chemotherapy or biological therapies rechallenge and intermittent treatment. Moreover, we will discuss the potential biological mechanisms of the observed results of early clinical trials.

## Introduction

Colorectal cancer (CRC) accounts for approximately three hundred thousand deaths worldwide every year. In metastatic CRC (mCRC), 5-year survival is only 6% worldwide, 11, 6% in US population and the identification of reliable prognostic factors in this disease has been an important focus of research in the last decade [1]. For decades fluoropyrimidines formed the backbone of treatment in mCRC. The relatively recent introduction of oxaliplatin, irinotecan and biologic therapies (Bevacizumab, Panitumumab and Cetuximab) allowed to reach the median overall survival of 23–24 months and up today monoclonal antibodies combined with standard chemotherapy are recommended for management of mCRC [2]. But the improvement in survival for mCRC patient led to two main outstanding issues: 1) there is a significant number of patients progressing beyond the third or fourth line of treatment still suitable for further therapy when enrollment into clinical trial is not possible. In this situation, the role of any therapy rechallenge (either chemotherapy alone, chemotherapy and biologic therapy or biologic therapy alone) is still not clear, particularly in patients who had previously responded, and if treatment choice is based on traditional dogma of primary and secondary resistance, rechallenge does not seem to be justified. 2) Prolonged intensive treatment is burdened from the high risk of cumulative toxicity, worsening in quality of life and a not well defined possibility of early acquired resistance.

According to a traditional dogma in medical oncology, a CRC patient is defined as resistant to treatment if the disease fails to respond (primary resistance) or initially responds and then progresses (secondary resistance) on a specific chemotherapy drug or regimen. Therefore, rechallenging patients’ disease with a drug or drugs to which their tumors are resistant seems to be inadvisable.

Recently two different strategies are emerging in mCRC treatment which seem to refute the traditional dogma of irreversible acquired resistance suggesting different possibilities to reverse or maintain the chemotherapy sensitiveness.

## Rechallenge therapy as a rescue possibility to reverse acquired resistance in highly pretreated mCRC patient

### Definition of rechallenge therapy

Rechallenge therapy is defined as reintroduction, after an intervening treatment, of the same therapy to which tumor has already proved to be resistant. To our knowledge, there are few evidences of mCRC sensitivity to any rechallenged therapy (Table 1).

**Table 1:** Definition of rechallenge therapy and intermittent therapy

**Definition of rechallenge therapy**	Reintroduction, after an intervening treatment, of the same therapy to which tumor has already proved to be resistant
**Definition of intermittent therapy**	Interruption of treatment without any evidence of tumor resistance in order to avoid cumulative toxicities and maintain a good quality of life and tumor sensitivity.

### Biological rationale and first preclinical evidences of anti-EGFR rechallenge efficacy

CRC is a complex disease involving many dysregulatory phenomena in a number of signal transduction pathways [3]. It has been shown that epidermal growth factor receptor (EGFR), a tyrosine kinase receptor belonging to the ErbB family, is overexpressed in 25%–80% of CRCs and plays a major role in its pathogenesis [4].

Subsequently, several clinical trials have demonstrated the therapeutic efficacy of antibodies targeting EGFR (cetuximab and panitumumab) in the treatment of CRC patients [5]. However, the overall response rate (ORR) to cetuximab or panitumumab based regimens is less than 30%, suggesting that primary resistance mechanisms are present in many cases [6-19]. The determination of Kirsten Rat sarcoma viral oncogene homologue (K-Ras) gene mutational status through different molecular techniques has recently became essential for the management of CRC patients as in other human neoplasia [20,21]. Several retrospective and prospective analysis showed that mutations K-Ras could justify primary resistance to anti-EGFR therapy [22-25], but molecular basis of secondary resistance to anti-EGFR therapy are not understood.

Previous studies suggest that K-Ras mutation is an early pathogenic step in colorectal cancer development and remain the same during tumor progression [26]. In fact, the same K-Ras mutations can be detected in most adenoma and in more than a half of the tumor adjacent mucosa [27]. One study provided first evidence that secondary K-Ras mutations do not occur during anti-EGFR therapy in CRC patients preserving a potential sensibility to cetuximab or panitumumab rechallenge [28]. Moreover a recent study from Baldus et al. evaluated K-Ras, BRAF and PI3K gene status into the primary tumor, comparing the tumor center and the invasion fronts showing that intratumoral heterogeneity of K-Ras, BRAF, and PIK3CA mutations was observed in 8%, 1%, and 5% of primary tumors, respectively [29].

### Conflicting clinical evidences of the activity of anti-EGFR therapy rechallenge

Given these evidences, a multicenter phase II prospective study investigated the activity of a rechallenge with a cetuximab-based therapy in 39 patients who first had a clinical benefit after a line of cetuximab plus irinotecan-based therapy, then a disease progression for which received a new line of chemotherapy and finally, after a further progression of disease, were re-treated with the same cetuximab plus irinotecan based therapy. Treatment holiday was not allowed. Median time to progression with first treatment with cetuximab was 10 months, the median interval time between last cycle of first cetuximab-based therapy and first cycle of the following cetuximab retreatment was 6 months. Moreover, ORR was 53.8% with 19 partial responses (48.7%) and 2 complete responses (5.1%). The median time to progression (TTP) was 6.6 months, stable disease (SD) was obtained in 35.9% of patients and progression in 4 (10.2%), and 18 patients (46.1%) showed the same type of response (SD, partial response or complete response) during cetuximab retreatment when compared with the response obtained during the first cetuximab-based therapy. Then stable disease lasting at least 6 months and partial response during the first cetuximab-based therapy have been demonstrated to predict clinical benefit after cetuximab retreatment [30].

Conversely, a subsequent phase II prospective study, including twenty patients treated with panitumumab after progression on prior cetuximab-based therapy, did not show any response to panitumumab being stable disease (no progression for at least two cycles) the best response in 45% of patients [31]. This study showed that panitumumab has a minimal effect after disease progression on cetuximab; however, no interval therapy or treatment holiday were permitted between cetuximab and panitumumab administration.

Diaz Jr et al. evaluated the variation of circulating tumor DNA (ctDNA) in serum of 24 patient receiving single-agent therapy with panitumumab. K-Ras mutations were recorded in 38% of cases between 5–6 months following treatment and mathematical modelling indicated that mutations were present in expanded subclones before the initiation of treatment. These results suggest that the emergence of KRAS mutations is a mediator of acquired resistance to EGFR blockade [32]. Consistently, another small study showed that point mutations of K-Ras are casually associated with the onset of acquired resistance to anti-EGFR therapy. In fact analysis of metastasis from ten patients who developed resistance to cetuximab or panitumumab showed the emergence of K-Ras mutant alleles were detectable in the blood months before the radiographic documentation of disease progression, and the in vitro model confirmed the hypothesis of continuing mutagenesis under the pressure of anti-EGFR therapy [33].

**Figure 1:**
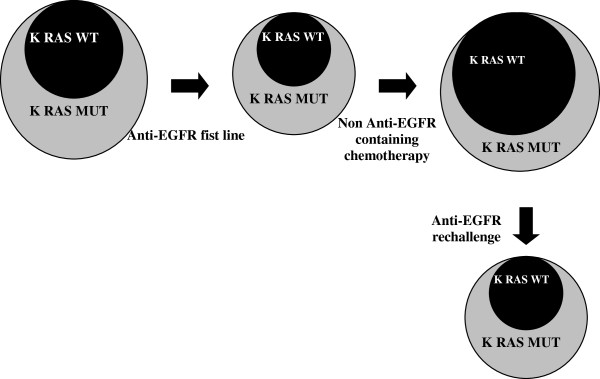
K-Ras WT clone restored during intervening chemotherapy allow the gain of new sensibility to anti-EGFR chemotherapy.

These studies underlined the possibility of late acquisition of K-Ras secondary mutations under anti EGFR therapy but still do not confute the possibility of a rechallenge. In fact an interval therapy after first progression to the anti-EGFR therapy could restore a partial sensitivity of tumor to the rechallenge by promoting the expansion of K-Ras wild-type clones returning, which will constitute the major part of the tumor mass at the time of a following progression of disease. A rescue through a cetuximab based new line therapy may then determine a further disease response (Figure 1).

In this sense an interval therapy based on a different treatment, which is not influenced by K-Ras status or is more efficacious in K-Ras mutated CRC, could facilitate the re-emersion of wt clones (Table 2).

**Table 2:** Biological and clinical data suggesting a possible role of rechallenge in management of mCRC

**Role of rechallenge in mCRC**	**K-ras status concordance and heterogeneity**	K-ras mutation is an early pathogenic step in colorectal cancer development and the possibility of late acquisition of K-Ras mutation is not clarified. The following therapy could allow K-Ras WT clone to re-predominate
	**Treatment holiday**	Holiday from a drug could allow reversion to a previous epigenetic profile. Moreover treatment holiday could facilitate recovery from cumulative toxicity induced by chemotherapy. To our knowledge few studies evaluated role of treatment holiday and they reported results.

An in vitro model suggested that K-Ras mutated cell lines are more sensitive to Oxaliplatin [34]. Consistently, a retrospective study evaluating K-Ras status in 90 patients treated with FOLFOX-6 as first-line or second-line treatment showing that PFS was longer in mutated K-Ras population than in wt K-Ras patients (10 vs 8 months, respectively; p = 0.001) [35].

### Clinical evidence of activity of standard chemotherapy rechallenge

The RE-OPEN phase II study assessed the efficacy of the re-introduction of oxaliplatin (administered in FOLFOX regimen) for 18 patients with metastatic colorectal cancer refractory to standard chemotherapy regimens including oxaliplatin, irinotecan and fluorouracil. Disease control rate (DCR) after 12 weeks was observed in seven patients (38.9%) [36].

## Treatment holiday and chemotherapy-free interval strategies

### Rationale

The introduction of biologic compounds in combination with standard chemotherapy in the treatment of mCRC has extended median overall survival of patients up to 2 years and beyond. Moreover a sequential treatment approach using all active agents can allow to reach long-term control of disease changing mCRC from an acute to chronic condition. In this new scenario, the quality of life and the avoidance of cumulative toxicity became one of the most important end point of mCRC management.

Several randomized phase III studies evaluated the role of chemotherapy in mCRC but most of them planned treatment to be continued until disease progression or development of intolerable toxicity. Starting from these evidences, mCRC patients will spend most of their remaining life receiving continuous anti-tumor therapy, with the associated toxic effects, periodic clinic visits detriment to quality of life.

Recently, some studies have investigated the role of intermittent chemotherapy in order to permit treatment holiday avoiding cumulative toxicity and preserving a good quality of life. Moreover, other new studies analyzed the role of biological agents (bevacizumab or cetuximab) given as an intervening therapy during chemotherapy holiday.

Most importantly, giving these therapies for a restricted period and then restart with or without evidence of disease progression in the interval is a potential method for reducing the emergence of acquired resistance to chemotherapy. In fact epigenetic instability belonging to tumoral mass might drive resistance under treatment selective pressure. It is therefore possible that an holiday from a drug could allow reversion to a previous epigenetic profile or could facilitate re-emersion of sensitive clones. To our knowledge few studies evaluated role of treatment holiday (or intermittent therapy) and chemotherapy free-interval (CFI).

### Studies evaluating efficacy and feasibility of chemotherapy administered in a stop-and-go strategy

A retrospective study analyzed reintroduction of FOLFOX in 29 patients affected by mCRC after a break in treatment or disease progression after another regimen. Six patients achieved an objective response, corresponding to a rate of 20.7%; among patients who received no intervening chemotherapy, the objective response rate was 31%, whereas for patients who received intervening chemotherapy the objective response rate was 12%. Five of the responses were observed among patients who had previously responded to FOLFOX treatment, whereas one response occurred in a patient who had previous progression. SD was achieved in 15 patients (52%), including seven patients (44%) who received no intervening chemotherapy and eight (62%) who received intervening chemotherapy. Clinical benefit was observed in 73% of cases, progression free survival (PFS) was 4.2 months, and OS was 9.7 months [37].

The OPTIMOX 1 study also assessed the role of reintroduction of oxaliplatin in a stop and go strategy. This study compared treatment with FOLFOX4 until progression with FOLFOX7 for 6 cycles, followed by maintenance with leucovorin–5-FU alone and FOLFOX7 reintroduction for a further 6 cycles. Six hundred twenty patients were enrolled, median PFS and OS were 9.0 and 19.3 months, respectively, in patients treated with FOLFOX4 compared with 8.7 and 21.2 months, respectively, in patients treated with FOLFOX7 in a stop-and-go strategy (P = not significant). Oxaliplatin was reintroduced in only 40.1% of the patients but achieved responses or stabilizations in 69.4% of these patients. Results show that ceasing oxaliplatin after 6 cycles, followed by leucovorin–5-FU alone, achieves RR, PFS, and OS equivalent to that with continuing oxaliplatin until progression or toxicity [38].

This data showed that treatment holiday is not associated with worsen outcome and delay presentation of cumutalive toxicity from oxaliplatin. However this trial do not assess the efficacy of oxaliplatin reintroduction after additional lines of therapy (ie, irinotecan and anti-EGFR or anti-VEGF therapy) and do not analyze the role of a real treatment holiday.

The OPTIMOX 2 phase II trial randomised 216 patients to receive fluorouracil maintenance between FOLFOX administration versus a treatment holiday. The primary objective was the duration of disease control (DDC), calculated as the sum of the duration of PFS induced with the initial FOLFOX therapy and with the subsequent reintroduction of FOLFOX. But most importantly, after induction of a response, metastases were allowed to progress back to baseline levels before FOLFOX was reintroduced.

It was observed that continuing treatment with a maintenance chemotherapy led to a longer PFS, compared with pausing treatment (8.7 months vs 6.9 months, P = 0.009) but overall survival data were not available [39,40]. DDC was almost identical in both arms (12.9 months vs 11.7 months, P not significant and duration of CFI seemed to depend on different clinical prognostic factors including Eastern Cooperative Oncology Group performance status, lactate dehydrogenase and alkaline phosphatase levels, number of metastatic sites. These data showed the possibility of identifying a favourable prognosis group which could benefit from an intermittent strategy.

The COIN phase III study randomized 1630 patients with untreated metastatic colorectal cancer to receive either continuous oxaliplatin and fluoropyrimidine combination (arm A), continuous chemotherapy plus cetuximab (arm B), or intermittent (arm C) chemotherapy. In arms A and B, treatment continued until development of progressive disease, cumulative toxic effects, or the patient chose to stop. In arm C, patients who had not progressed after six cycles of chemotherapy started a treatment holiday until evidence of disease progression, when the same treatment was restarted. Median survival was 15.8 months in arm A vs 14.4 months in arm C (hazard ratio 1.084, 80% CI 1.008–1.165). In the per-protocol population, more patients on continuous than on intermittent treatment had grade 3 or worse haematological toxic effects (15% vs 12%), whereas nausea and vomiting were more common on intermittent treatment (2% vs 8%). Other grade 3 or worse toxicities (such as peripheral neuropathy and hand–foot syndrome) were more frequent on continuous than on intermittent treatment [41].

### Studies evaluating efficacy and feasibility of biological therapy administered during chemotherapy-free interval

The NORDIC VII multicenter phase III trial randomly assigned 571 previously untreated patients to receive the standard Nordic FLOX, cetuximab and FLOX, or cetuximab combined with intermittent FLOX. Median PFS was 7.9, 8.3, and 7.3 months for the three arms, respectively (not significantly different). But OS was almost identical for the three groups (20.4, 19.7, 20.3 months, respectively), and confirmed RR were 41%, 49%, and 47%, respectively [42].

The phase II COIN-B trial randomized patients to receive cetuximab and chemotherapy (Arm D) in an intermittent schedule versus intermittent chemotherapy with continuous cetuximab administration (Arm E). Upon RECIST progression on either arm, the same chemotherapy plus cetuximab was restarted and continued until progression. Continuous cetuximab administration as maintenance was associated with a longer CFI and longer PFS (5,1 and 13,7 months respectively vs 3,7 and 12 months in the arm D) [43].

The MACRO TTD phase III trial randomized 480 previously untreated mCRC patients to receive 6 cycles of bevacizumab and Xelox followed by Xelox and bevacizumab (arm A) or bevacizumab alone (Arm B). There were not statistically significant differences in PFS and OS between the 2 arms [44]. This study confirmed the efficacy of a maintenance therapy with bevacizumab after a predefined period of chemotherapy induction but did not investigated the role of bevacizumab maintenance in a stop-and-go strategy with a subsequent reintroduction of the same chemotherapy when disease progression occurs.

In the ongoing AIO study, maintenance treatment with capecitabine or 5-FU/folinic acid and bevacizumab is compared with bevacizumab alone or no maintenance treatment in subjects with inoperable and non-progressive metastatic colorectal cancer after first line induction treatment for 24 weeks with a fluoropyrimidine-, oxaliplatin- and bevacizumab-based chemotherapy. Reinduction treatment will be done in case of progression (Table 3).

**Table 3:** Clinical evidences evaluating different strategies for treatment of mCRC

**EGFR therapy rechallenge**	- A multicenter phase II prospective study confirmed the activity of cetuximab rechallenge plus irinotecan-based therapy after an intervening chemotherapy [30]
	- A phase II prospective study did not show any response to panitumumab administrated after progression on prior cetuximab-based therapy [31]
**Chemotherapy stop-and go strategy**	- OPTIMOX 1 study shows that ceasing oxaliplatin after 6 cycles, followed by leucovorin–5-FU alone, achieves RR, PFS, and OS equivalent to that with continuing oxaliplatin until progression or toxicity [38]
	- OPTIMOX 2 study shows that continuing treatment with a maintenance chemotherapy led to a longer PFS, compared with pausing treatment [39]
	- COIN study did not show a non inferiority of chemotherapy free interval versus continuous treatment but treatment holiday significantly reduced cumulative toxic effects, and improved quality of life [41]
**Biological treatment of chemotherapy-free interval**	- NORDIC VIII phase III trial showed that cetuximab maintenance do not improve survival data comparing to intermittent treatment [42].
	- COIN B phase II trial showed that cetuximab maintenance significantly improved chemotherapy free interval and PFS [43].
	- MACRO TTD phase III trial confirmed the efficacy of a maintenance therapy with bevacizumab after a predefined period of chemotherapy induction [44].
	- CAIRO 3 phase III trial showed that bevacizumab and de-escalated chemotherapy maintenance administrated after chemotherapy and bevacizumab induction significantly improves OS comparing to a treatment holiday strategy [45].

These studies do not allow a clear indication on what is the best option between treatment holiday (defined as pause from all treatment) and chemotherapy-free interval with a period of maintenance therapy, and more prospective trial are warranted.

## Conclusions

The role of rechallenge therapy in third-line or fourth-line setting in mCRC is not defined but it could be a possibility for fit patients who do not have any other valid options.

Few clinical studies evaluated the role of targeted therapies rechallenge and up to date there are no convincing predictive factors suggesting which drug should be readministered. This choice should be based on several reasonable factors: best response to prior treatment before progression (prolonged stable disease, partial response or complete response), residual toxicity (especially in case of oxaliplatin reintroduction), duration of treatment holiday.

In our opinion, intermittent treatment could be an important strategy in management of mCRC patient when there is not the purpose of gaining an important tumour shrinkage, for avoiding cumulative toxicity and for maintaining chemotherapy sensitiveness even if there is not a clear evidence in prolonging OS compared to the intensive treatment.

Moreover, few clinical studies assessed the role of rechallenge in the era of targeted therapy and no studies evaluated the activity of bevacizumab as a rechallenge therapy (both as a monotherapy or in combination with standard chemotherapy) so far. However, it has been demonstrated that targeted therapy could enhance sensitivity to both chemotherapy and radiotherapy [46]. Brite and TML study showed a benefit in the use of bevacizumab beyond disease progression. However, in this case, we cannot regard to bevacizumab administration as a real rechallenge, as there was no treatment interruption after disease progression or any intervening therapy. Further clinical studies should enquire the role of bevacizumab retreatment and the importance of angiogenesis control in heavily pretreated mCRC patients as a possible mechanism of restoring sensitivity to re-administration of standard chemotherapy. However this evaluation should take into account several new evidences: 1) recent studies proved the efficacy of bevacizumab as maintenance therapy without any interruption, 2) there are data suggesting the efficacy of bevacizumab beyond progression [47-49]; 3) preclinical evidences demonstrated that there is a reversible tumor growth acceleration following bevacizumab interruption [50] 4) a phase III trial showed that bevacizumab and de-escalated chemotherapy maintenance administrated after chemotherapy and bevacizumab induction significantly improved OS comparing to a treatment holiday strategy (21.7 vs 17.9 months, p = 0,02) [45]. So a real standard strategy regarding bevacizumab administration through several lines of treatment of mCRC patients is still not defined.

In this sense, to date, there are no phase III trial comparing the bevacizumab rechallenge strategy (bevacizumab readministration after a treatment holiday) with bevacizumab-alone maintenance and with a de-escalated chemotherapy and bevacizumab maintenance. The ongoing AIO study could suggest which is the better strategy applying to bevacizumab administration.

Moreover, clinical trials evaluating predictive factors of response to chemotherapy and biologic agents rechallenge or to intermittent therapies are warranted in order to select patients, avoid possible side effect and useless waste of resources. In addition, randomized trials should be performed to understand the clinical impact of rechallenge and intermittent treatment strategies in advanced CRC patients.

## Competing interests

The authors declare that they have no competing interests.

## Authors’ contributions

GT has developed the conclusions paragraph and reviewed the manuscript. MI collected data from literature and wrote the manuscript. AMF collected data from literature and wrote the manuscript. BV collected data from literature and wrote the manuscript. DS has developed the introduction paragraph and reviewed the manuscript. All authors read and approved the final manuscript.
